# Do Children With Congenital Zika Syndrome Have Cerebral Palsy?

**DOI:** 10.9745/GHSP-D-21-00575

**Published:** 2022-10-31

**Authors:** Alessandra Carvalho, Egmar Longo, Cristiana Nascimento-Carvalho, Nayara Argollo, Kátia Edni Coelho, Aline Sampaio, Carlos Brites, Rita Lucena

**Affiliations:** a SARAH Network of Rehabilitation Hospitals, Salvador, Bahia, Brazil.; b Trairi Health Sciences Faculty (FACISA), Federal University of Rio Grande do Norte, Santa Cruz, Rio Grande do Norte, Brazil.; c Federal University of Bahia, Salvador, Bahia, Brazil.

## Abstract

As researchers and practitioners, we have an important role in educating families of children with brain damage caused by Zika virus infection on how a cerebral palsy diagnosis can empower them with more information and enable better access to care and intervention services.

## Key Messages

- Children with congenital Zika syndrome should consistently be described as having cerebral palsy when meeting the criteria for this diagnosis.
- Having access to cerebral palsy–based services will help families and health care professionals in their rehabilitation approaches for children with congenital Zika syndrome.

Resumo em português no final do artigo.

## INTRODUCTION

According to the Definition and Classification of Cerebral Palsy, April 2006, cerebral palsy (CP) refers to^1^:

*a group of permanent disorders of movement and posture, causing activity limitation, that are attributed to non-progressive disturbances that occurred in the developing fetal or infant brain. In addition to the motor symptoms, disturbances of sensation, perception, cognition, communication, behaviour, epilepsy, and secondary musculoskeletal abnormalities may also be present.*

Intrauterine infections, including congenital infections, are reported to be the identified etiology in 5%–10% of cases of CP, with congenital cytomegalovirus being the most common viral infection related to this diagnosis.^2^

In 2015, an outbreak of the Zika virus was reported in Brazil, followed by a significant increase in the prevalence of congenital microcephaly and neurological abnormalities in children born to mothers infected with the Zika virus during pregnancy.^3^ The causal link between those events was confirmed, and vertical transmission of the virus was established for the first time.^4^^,^^5^ The affected children were soon described as having congenital Zika syndrome (CZS). The classic phenotype comprised severe microcephaly and partly collapsed skull, typical neuroimaging features (cerebral atrophy and subcortical calcifications), ocular abnormalities (macular scarring and focal pigmentary retinal mottling), arthrogryposis, and early severe hypertonia.^6^ This classic clinical picture was reported to be present in 5%–14% of the infected newborns.^7^ A thorough analysis of the literature showed that the clinical and neuroradiological features could be present in a spectrum of severity, even though the majority of the descriptions were of significantly affected children who had several comorbidities, such as epilepsy, hypertonia, dysphagia, and visual and auditory abnormalities. Despite having a similar clinical picture, CP was not clearly mentioned as a common diagnosis in the majority of the studies on children born to mothers infected with the Zika virus during pregnancy.^8^

Despite CP and CZS having a similar clinical picture in children, most studies did not mention CP as a diagnosis in children with CZS.

## A NEW ETIOLOGY OF CP EMERGES

Since its first recognition, CZS—this new etiology of CP—has been more commonly diagnosed than CP in clinical and epidemiological scenarios, which is demonstrated by the small number of publications that mention a CP diagnosis,^8^ the stronger identification with microcephaly or Zika (and not CP) adopted by families of affected children, and even the lack of recognition of CP by some health care workers and rehabilitation providers who care for those patients. This focus on the etiology made sense at the beginning of the microcephaly epidemic because the scientific community was facing an unknown and unexpected situation. However, now more than 5 years after the first reports, it is time to broaden the perspective and combine efforts to improve the support for children with neurodevelopmental impairments that are part of the CP spectrum, regardless of the etiology.

We would like to highlight the idea that children with brain impairments caused by the Zika virus and a clinical diagnosis compatible with CP should consistently be recognized under the umbrella term of CP, which is a heterogeneous clinical descriptive condition rather than an etiologic diagnosis. We argue that, as in the broad definition of CP, children with brain damage related to Zika virus infection have been described by the literature as having a motor disorder, with difficulties in gross and fine motor functions as core features but also with other comorbid developmental disorders, such as sensory issues, intellectual disability, language impairments, and epilepsy.^8^ As with other classic intrauterine infections (e.g., rubella, toxoplasmosis, and cytomegalovirus), the underlying neuropathological event occurred during intrauterine life and is no longer active at the time of diagnosis, with a disruption of brain structure and function, leading to permanent but nonprogressive brain impairment.^9^ The functional status and dynamic needs of children with CZS are very similar to those of other children with CP, with the majority classified as Gross Motor Function Classification System IV or V.^8^^,^^10^ Even children who have an associated diagnosis of arthrogryposis related to CZS could be described as having CP if all the criteria for this diagnosis are present. It is worth noting that arthrogryposis is an umbrella term that includes a variety of causes (e.g., abnormalities in the central nervous system), and the literature has provided examples of the association between arthrogryposis and CP.^11^^,^^12^

Finally, we suggest that the etiologic diagnosis idea should not be replaced or understated, but rather, a complementary description of the condition should be used that could be helpful for parents and health care professionals in their rehabilitation approaches.

## WHY DOES THIS DIAGNOSIS MATTER?

The clear designation of a CP diagnosis and its relationship with CZS should be better explained to the families and acknowledged by health care providers and the scientific community. When we think about the pillars of evidence-based rehabilitation practice, our ability to accomplish this paradigm shift would help in several ways.

1. From the children’s and families’ perspectives, having this designation would represent an opportunity to improve communication between health care and rehabilitation providers and the families regarding prognosis and therapeutic options. For example, the possibility to use the Gross Motor Function Classification System as a common language to understand the prognosis and establish therapeutic goals would be highly beneficial because this classification should be used only in children with a diagnosis of CP.^13^
2. From the rehabilitation professional’s perspective, having this designation would allow the potential use of a range of evidence-based rehabilitation approaches previously known for CP.
3. From the scientific literature perspective, this designation would allow recognition of opportunities to use validated standardized tools for CP and to report important clinical and epidemiological data. Aside from treatment and rehabilitation approaches, other areas of interest, such as knowledge translation, advocacy, as well as public policies aimed at primary prevention, would also benefit from this concept of children with CZS being acknowledged within the broader CP community.

**Figure:**
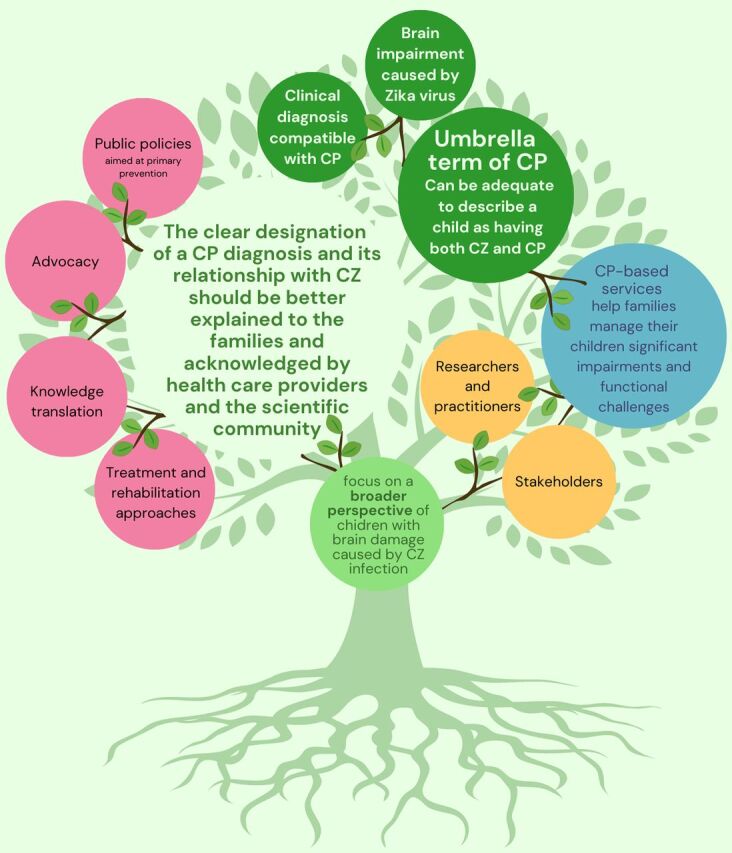
Empowering families of children with congenital Zika syndrome with a diagnosis of cerebral palsy will enable better access to rehabilitation and care.

**Figure:**
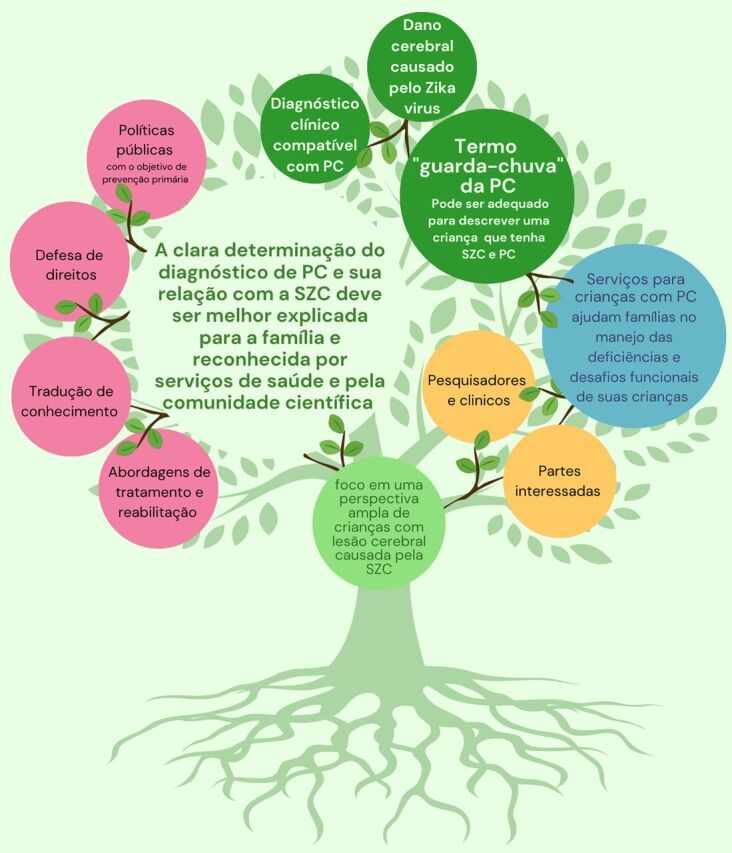
O empoderamento das famílias das crianças com síndrome Zika congénita com um diagnóstico de paralisia cerebral permitirá um melhor acesso à reabilitação e aos cuidados.

Since CZS has emerged only recently as a relevant cause of CP, especially in places where the 2015 epidemic hit harder, there have been few studies about therapeutic and rehabilitation approaches focused on the use of the International Classification of Function, Disability and Health framework, especially considering gold standard participation outcomes.^14^ Certainly, the considerable number of evidence-based strategies that are used in CP could be applied to CZS, which would support the therapy services and increase the options of care for those children. Brazil, as well as other low- and middle-income countries, have already had difficulties in implementing evidence-based interventions for children with CP, a consequence of the lack of training opportunities across the country.^15^^,^^16^ Thus, considering CZS as a separate condition would pose an even greater burden to the challenge of providing the best evidence-based care for these children.

A considerable number of evidence-based strategies that are used in CP could be applied to CZS, which would support the therapy services and increase the options of care for those children.

## CONCLUSION

CP is a clinical description and not a disease, which means that it can be adequate to describe a child as having both CZS and CP. As such, CP-based services should be appropriate to help families manage their children’s significant impairments and the Portuguese version of the photo with the new version in file challenges, which does not take away the Zika etiology and this population’s specificities. Considering that CZS is a condition known for only 5 years, it is worth continuing the follow-up of affected cohorts worldwide. As researchers and practitioners, we have an important role in helping to educate these stakeholders to look beyond a pure etiologic paradigm and focus on a broader perspective of children with brain damage caused by Zika virus infection. This will ultimately enable and empower their families to have more information and better access to care and intervention services.

## Translation

**Em Português**

**Crianças Com Síndrome da Zika Congênita apresentam Paralisia Cerebral?**

Mensagens-chave:

- Crianças com síndrome da Zika congênita devem ser consistentemente descritas com o diagnóstico de paralisia cerebral, se preencherem critérios para tal diagnóstico.
- Serviços de reabilitação para paralisia cerebral são apropriados para auxiliar famílias e profissionais de saúde nas abordagens de reabilitação de crianças com infecção congênita pelo vírus Zika.

**Peer Reviewed**

**First published online:** October 14, 2022.

## References

[1] Rosenbaum P Paneth N Leviton A. A report: the definition and classification of cerebral palsy April 2006. Dev Med Child Neurol Suppl. 2007;49(109):8–14. 10.1111/j.1469-8749.2007.tb12610.x. 17370477

[2] Hermansen MC Hermansen MG. Perinatal infections and cerebral palsy. Clin Perinatol. 2006;33(2):315–333. 10.1016/j.clp.2006.03.002. 16765727

[3] Pan American Health Organization (PAHO), World Health Organization (WHO). Epidemiological Update: Neurological Syndrome, Congenital Anomalies, and Zika Virus Infection. PAHO/WHO; 2016. Accessed October 4, 2022. https://www.paho.org/hq/dmdocuments/2016/2016-jan-17-cha-epi-update-zika-virus.pdf

[4] de Araújo TVB Ximenes RAA Miranda-Filho DB. Association between microcephaly, Zika virus infection, and other risk factors in Brazil: final report of a case-control study. Lancet Infect Dis. 2018;18(3):328–336. 10.1016/S1473-3099(17)30727-2. 29242091 PMC7617036

[5] Brasil P Pereira JP Jr Moreira ME. Zika virus infection in pregnant women in Rio de Janeiro. N Engl J Med. 2016;375(24):2321–2334. 10.1056/NEJMoa1602412. 26943629 PMC5323261

[6] Moore CA Staples JE Dobyns WB. Characterizing the pattern of anomalies in congenital Zika syndrome for pediatric clinicians. JAMA Pediatr. 2017;171(3):288–295. 10.1001/jamapediatrics.2016.3982. 27812690 PMC5561417

[7] Musso D Ko AI Baud D. Zika virus infection — after the pandemic. N Engl J Med. 2019;381(15):1444–1457. 10.1056/NEJMra1808246. 31597021

[8] Carvalho A Sales HF Ventura P Gnoatto-Medeiros M Brites C Lucena R. The neurodevelopmental spectrum of congenital Zika infection: a scoping review. Dev Med Child Neurol. 2020;62(12):1356–1362. 10.1111/dmcn.14675. 32931050

[9] Petribu NCL Aragao MFV van der Linden V. Follow-up brain imaging of 37 children with congenital Zika syndrome: case series study. BMJ. 2017;359:j4188. 10.1136/bmj.j4188. 29030384 PMC5639438

[10] Melo A Gama GL Da Silva Júnior RA. Motor function in children with congenital Zika syndrome. Dev Med Child Neurol. 2020;62(2):221–226. 10.1111/dmcn.14227. 30945276

[11] Engel S Ferrara G. Obstetric outcomes in women who sustained a spinal cord injury during pregnancy. Spinal Cord. 2013;51(2):170–171. 10.1038/sc.2012.125. 23247014

[12] Dahan-Oliel N Cachecho S Barnes D. International multidisciplinary collaboration toward an annotated definition of arthrogryposis multiplex congenita. Am J Med Genet C Semin Med Genet. 2019;181(3):288–299. 10.1002/ajmg.c.31721. 31282072 PMC6771513

[13] Towns M Rosenbaum P Palisano R Wright FV. Should the Gross Motor Function Classification System be used for children who do not have cerebral palsy? Dev Med Child Neurol. 2018;60(2):147–154. 10.1111/dmcn.13602. 29105760

[14] Longo E de Campos AC Schiariti V. Zika virus after emergency response: can the ICF guide rehabilitation of children with microcephaly? Pediatr Phys Ther. 2019;31(4):370–372. 10.1097/PEP.0000000000000647. 31568386

[15] Benfer KA Novak I Morgan C. Community-based parent-delivered early detection and intervention programme for infants at high risk of cerebral palsy in a low-resource country (Learning through Everyday Activities with Parents (LEAP-CP): protocol for a randomised controlled trial. BMJ Open. 2018;8(6):e021186. 10.1136/bmjopen-2017-021186. 29934387 PMC6020941

[16] Longo E de Campos AC Palisano RJ. Let’s make pediatric physical therapy a true evidence-based field! Can we count on you? Braz J Phys Ther. 2019;23(3):187–188. 10.1016/j.bjpt.2018.10.011. 30420270 PMC6531638

